# More absolute moderate-to-vigorous physical activity is associated with better health-related quality of life in outpatients with an acquired brain injury

**DOI:** 10.3389/fresc.2024.1427446

**Published:** 2024-12-06

**Authors:** Emily E. MacDonald, Liam P. Pellerine, Katerina E. Miller, Ryan J. Frayne, Myles W. O’Brien

**Affiliations:** ^1^ Department of Neuroscience, Dalhousie University, Halifax, NS, Canada; ^2^ Division of Kinesiology, Faculty of Health, School of Health and Human Performance, Dalhousie University, Halifax, NS, Canada; ^3^ Acquired Brain Injury NeuroCommons, Nova Scotia Health Authority, Halifax, NS, Canada; ^4^ Department of Medicine, Université de Sherbrooke, Sherbrooke, QC, Canada; ^5^ Centre de Formation Médicale du Nouveau-Brunswick, Université de Sherbrooke, Moncton, NB, Canada

**Keywords:** physical activity intensity, relative physical activity, 6-min walk test, quality of life, brain injured patients

## Abstract

Health-related quality of life (HRQoL) is a patient-perceived measure of physical, social, and emotional health. Acquired brain injury (ABI) occurs due to damage to the brain after birth. Individuals with an ABI typically present with reduced HRQoL and require additional support to maintain their HRQoL. Although structured exercise training has been shown to improve HRQoL in individuals with ABI, there is little research on habitual, real-world activity. Most activity research characterizes moderate-to-vigorous physical activity (MVPA) in absolute terms; however, relative physical activity levels have been promoted for research in clinical populations. We tested whether longer MVPA durations, measured in absolute/relative levels, are associated with higher HRQoL in outpatients with ABIs. In total, 26 adults (54 ± 13 years, 16 females) with ABI completed the Quality of Life After Brain Injury questionnaire, a 6-min walk test (a measure of aerobic fitness; 490 ± 105 m), and wore an activPAL device 24 h/day for 7 days. Participants had an average HRQoL score of 53.4 ± 15.0 (out of 100), with 20 of 26 showing impaired HRQoL (score <60). Absolute MVPA (74.6 ± 91.0 min/week, *b* = 0.09, *p* = 0.03) was associated with HRQoL, whereas total physical activity (565.7 ± 264.8 min/week, *p* = 0.47), light physical activity (LPA; 491.1 ± 224.3 min/week, *p* = 0.98), and step count (5,960 ± 3,037 steps/day, *p* = 0.24) were not. Neither relative LPA (521.4 ± 244.9) nor relative MVPA (33.5 ± 34.9 min/week) were associated with HRQoL (both *p* values > 0.14). Targeting more absolute MVPA, but not necessarily relative MVPA, may be an effective strategy for interventions aiming to improve HRQoL in individuals with ABI.

## Introduction

Acquired brain injury (ABI) occurs when brain damage is sustained after birth (1). ABI is typically categorized into two types: traumatic brain injury (TBI) and non-traumatic brain injury (NTBI) (2). TBI results from external forces, such as a concussion or motor vehicle accident, whereas NTBI arises when internal factors, like brain tumors or strokes, damage brain tissue (3). ABI commonly impairs neurological, physical, cognitive, and behavioral functions, with the severity of the deficit depending on the injury (4). Thus, individuals with ABI require additional support and resources to maintain their quality of life. Health-related quality of life (HRQoL) is a subjective, patient perception-focused measurement of physical, social, and emotional health (5). HRQoL is used not only as an indicator of overall health and the burden of disease on quality of life (6), but also for evaluating treatment methods and lifestyle adaptations (7). HRQoL measures can be either generic (e.g., SF 36-Item Short Form Survey) or disease-specific [Quality of Life After Brain Injury (QOLIBRI)] (8), with generic questionnaires being less applicable to clinical populations, specifically those with ABI (9), because of the unique physical, cognitive, emotional, and behavioral impairments experienced by this population (7). Understanding the impact of lifestyle adaptations on improving HRQoL is of particular importance when studying individuals with ABI.

The Canadian 24-h movement guidelines recommend that adults engage in at least 150 min of moderate-to-vigorous physical activity (MVPA) per week (10). Engaging in MVPA is associated with reduced risk of all-cause mortality, cardiovascular disease, and cancer mortality (11) and improved cognitive function (12). In populations of individuals with ABI, the duration of physical activity is consistently lower than the physical activity guideline recommendations (13). In a review of 26 physical activity monitoring studies, it was observed that accelerometry-based step counts were 50% lower in people with a stroke compared to those in aged-matched controls (14). In addition, an increase in self-reported and objectively measured physical activity in individuals post-stroke was associated with improved HRQoL, as assessed by the SF-36 (15). A systematic review of the effects of exercise interventions in individuals with ABI suggests that exercise, regardless of type (e.g., aerobic, resistance), improves HRQoL, as determined by the SF-36 (16). A non-randomized control trial of the Physical Activity and Sport for Acquired Brain Injury program demonstrated improvements in the physical and mental domains of HRQoL (17). Collectively, these studies emphasize that engaging in exercise interventions, specifically, self-reported physical activity, leads to improvements in HRQoL, indicating that such interventions should be a key component of care for outpatients with ABI. The continued use of the SF-36 highlights the need for more research using disease-specific instruments to determine whether these HRQoL benefits are consistently observed with such measurements. In addition, there is limited understanding of habitual, real-world physical activity in outpatients with ABI, as previous research has focused on structured exercise interventions. The reliance on self-report questionnaires for physical activity, especially when there are increased self-reported errors in patients with ABI (18), indicates the need for more research using objectively measured habitual physical activity.

Traditionally, physical activity has been characterized in terms of absolute activity intensity thresholds, depending on metabolic equivalents of the task (METs; a multiple of resting energy expenditure), with light physical activity (LPA) being any activity <3 METs and MVPA being classified as ≥3 METs (19). However, it is recommended by both the Canadian Society for Exercise Physiology (CSEP) and the American College of Sports Medicine (ACSM) that physical activity intensities be individualized to aerobic fitness, with MVPA defined as activity above 40% of maximal METs (MET_max_) (20, 21). More aerobically fit individuals typically meet the absolute guidelines for MVPA more readily than those with lower fitness levels (22). Given patients with ABI typically have lower aerobic fitness than the general population (23), it may be of particular importance to consider relative physical activity intensities that scale MVPA in relation to their maximal aerobic fitness.

The purpose of this study was to evaluate the relationship between physical activity and HRQoL in outpatients with ABI. It is hypothesized that increased time spent in both absolute and relative MVPA would be associated with higher HRQoL, as assessed by the ABI-specific QOLIBRI, in outpatients with ABI.

## Materials and methods

### Study design

Participants were recruited from an ABI Rehabilitation Day program at the NeuroCommons center in Bedford, Nova Scotia. Each participant wore an activPAL device for 24 h/day over 7 days. Participants were then asked to complete the QOLIBRI questionnaire to assess HRQoL. Finally, the participants completed the 6-min walk test (6MWT) (see below).

### Participants

In total, 26 adults (54 ± 13 years, 16 females) from the Nova Scotia Health's ABI Rehabilitation Day program between August 2022 and September 2023 were included. The sample included 16 individuals with NTBI (e.g., stroke) and 10 with TBI (e.g., concussion; 8 of 10 had a concussion). Participants were included if they were >18 years of age, scored >90 on the Functional Independence Measure, and could safely engage in MVPA. Of an original 30 participants, 4 were excluded due to incomplete or unusable physical activity data resulting from software issues or user error (e.g., a participant removed the monitor before the collection period ended) (*n* = 3) or because the HRQoL questionnaire was not completed (*n* = 1). Based on a large effect size (*f*^2^ = 0.35), a linear regression model indicated that a minimum of 25 participants were needed, assuming a two-tailed test with *α* = 0.05 and *β* = 80% power [G*Power, v3.1 (24)]. Research ethics approval was obtained from the Nova Scotia Health's Research Ethics Board (REB #39067), and all participants provided informed consent before data collection.

### Health-related quality of life

HRQoL is a subjective, patient perception-focused measurement of physical, social, and emotional health (5). The QOLIBRI is a validated instrument for measuring HRQoL in individuals with ABI (8). It is a 37-item scale with six subsections: cognition, emotion, daily autonomy, social relationships, mental impairments, and physical impairments, with responses recorded on a five-point Likert scale. The responses for Parts A–D of the QOLIBRI questionnaire were scored numerically (e.g., “not at all” = 0, “very” = 5), while responses for Parts E and F were inversely scored (e.g., “not at all” = 5, “very” = 0). The mean score for each section was calculated (e.g., sum of section A values/number of questions in section A), and the six section means (A–F) were then averaged to calculate a total mean score (sum of section mean scores/6). The total mean (e.g., 3.8 out of 5) was converted to a 0–100 scale (0 = no HRQoL, 100 = perfect HRQoL). This conversion was done by subtracting one from the total mean and multiplying by 25 [e.g., (3.8 − 1) × 25 = 70). A higher QOLIBRI score indicates better HRQoL, with a threshold score of <60/100 indicating low or impaired HRQoL (25).

### Six-min walk test

The 6MWT is a validated method for estimating maximal aerobic fitness (VO_2max_) in individuals with ABI (26). A track was set up with two plastic cones placed 30 m apart, marking the points at which participants were to turn 180°. Participants were asked to walk around the cones as many times as possible for 6 min. Participants were given scripted verbal encouragement every minute. The final distance was recorded to the nearest centimeter. Height and weight were measured using a calibrated stadiometer and a physician's scale (Health-O-Meter, McCook, IL, USA) to the nearest 0.5 cm and 0.1 kg, respectively. Body mass index was then calculated as $\;\mathrm{body}\;\mathrm{mass}\;(\mathrm{kg})/\mathrm{height}\;{\left(m\right)}^{2}\;$. VO_2max_ was estimated using the following equation:

$\begin{array}{r} 59.44-3.83\;\times \;\mathrm{sex}\;(1:\;\mathrm{males};\;2:\;\mathrm{females})-0.56\;\times \;\mathrm{age}\;\\ \left(\mathrm{years})-0.48\;\times \;\mathrm{BMI}\;(\mathrm{kg}/m^{2})\;+\;0.04\;\times \;\mathrm{the}\;6\mathrm{MWT}\;(m\right) \end{array}$ (27).

### Free-living activity monitoring

The activPAL inclinometer (V3 or V4, Pal Technologies Ltd., Glasgow, UK) was used to objectively measure physical activity and sedentary time. The activPAL is a validated device for assessing free-living posture (28) and physical activity (29). All participants wore the activPAL for 24 h/day over 7 days, following previous wear time recommendations (30). The activPAL was waterproofed and secured using a nitrile finger cot and transparent medical dressing to the midline of the right thigh, one-third of the way between the hip and knee (31).

Raw activPAL data were exported into the PAL analysis software (version 5.8.5) for initial data processing, which produced a range of activity summaries. Further processing of these summaries was conducted using a customized MATLAB program (MathWorks, Portola Valley, CA, USA), that calculated daily averages for time awake, standing time, and sedentary time. An additional LabVIEW program (National Instruments, Austin, TX, USA) determined time spent in each physical activity intensity via step rate thresholds determined based on body mass index (32). Time spent above step rate thresholds corresponding to LPA and MVPA were determined (see below).

### Absolute and relative physical activity

Absolute MVPA corresponds to time spent in >3 METs. Relative physical activity thresholds were calculated using >40% of MET_max_ based on the 6MWT-determined maximal aerobic fitness. For older adults (>55 years old), VO_2max_ was divided by 2.7 to calculate MET_max_, while for younger adults (≤55 years old), VO_2max_ was divided by 3.5 to determine MET_max_.

The step rate corresponding to absolute and relative MVPA was calculated using the height of the individual for younger adults according to the following equation:

$\begin{array}{r} \mathrm{Step}\;\mathrm{rate}=73.490-(0.513\times \mathrm{height})+(59.867\times \mathrm{METs})-\\ \left(8.500\times \mathrm{MET}s^{2})+(0.436\times \mathrm{MET}s^{3}\right) \end{array}$,

while the step rate for older adults was calculated using BMI according to the following equation:

$\begin{array}{r} \mathrm{Step}\;\mathrm{rate}=\text{–}84.321+(91.209\times \mathrm{METs})-(12.968\times \mathrm{MET}s^{2})+\\ \left(0.772\times \mathrm{MET}s^{3})+(2.211\times \mathrm{BMI})-(0.549\times \mathrm{BMI}\times \mathrm{METs}\right) \end{array}$ (33).

### Statistical analysis

The relationships between each physical activity intensity (LPA and MVPA), both in absolute and relative intensity terms and HRQoL were analyzed using linear regressions. Age and physical fitness (via the 6MWT) are known factors that can affect the physical activity level of an individual (34). Accordingly, the covariates included for comparing absolute physical activity, sedentary time, standing time, and step count with HRQoL were age and 6MWT. Analyses using *relative* physical activity intensity only included age as a covariate due to the integration of 6MWT into the calculation of relative intensity thresholds. The relationship between age (via Pearson's correlation), sex (via independent samples *t*-test), and ABI type (via independent samples *t*-test) with HRQoL was determined. All statistics were completed using SPSS version 28.0 (IBM, NY, USA). Multicollinearity was assessed using variance inflation factors, all of which were less than the standard threshold of 10 (all, <1.4). Statistical significance was accepted as *p* < 0.05. All data are presented as mean  ± standard deviations.

## Results

Participants included in this study had a mean age of 54 ± 13 years, a body mass index of 30.4 ± 7.6 kg/m^2^, and an average 6MWT distance of 390 ± 105 m (Table 1). Participants engaged in absolute LPA for 491.1 ± 224.3 min/week and in MVPA for 74.6 ± 91.0 min/week. Relative LPA averaged 521.4 ± 244.9 min/week, while relative MVPA averaged 33.5 ± 34.9 min/week. Participants had an average HRQoL score of 53.4 ± 15.0 (out of 100). In this sample, 77% of participants (*n* = 20/26) had an impaired HRQoL score of <60/100 (25). Scores on each individual section of the QOLIBRI are listed in Table 2. Age (*p* = 0.29), sex (*p* = 0.07), and ABI type (*p* = 0.39) were not associated with HRQoL. In addition, there were no differences in age (*p* = 0.18) or sex distribution (*p* = 0.68) between those with TBI and those with NTBI.

**Table 1:** Participants’ descriptive characteristics, habitual posture, and physical activity outcomes.

Variable	Participant (*n* = 26)
Age (years)	54 ± 13 (24–72)
Acquired brain injury (TBI, NTBI)	10, 16
Sex (male participants, female participants)	10, 16
Height (cm)	164.5 ± 8.6 (146.0–181.0)
Weight (kg)	82.8 ± 24.7 (50.0–169.0)
Body mass index (kg/m^2^)	30.4 ± 7.6 (21.4–60.6)
6MWT (m)	490 ± 105 (187–723)
Step count (steps/day)	5,960 ± 3,037 (757–14,793)
Sedentary time (h/day)	10.6 ± 2.4 (5.8–16.1)
Standing time (h/day)	4.7 ± 2.1 (0.7–9.0)
Absolute LPA (min/week)	491.1 ± 224.3 (93.0–1,097.1)
Absolute MVPA (min/week)	74.6 ± 91.0 (0.4–344.9)
Relative LPA (min/week)	521.4 ± 244.9 (24.7–1,294.3)
Relative MVPA (min/week)	33.5 ± 34.9 (0.5–120.6)
Absolute MVPA step rate threshold (steps/min)	110.6 ± 4.3 (105.5–127.6)
Relative MVPA step rate threshold (steps/min)	115.7 ± 14.2 (73.3–140.4)

TBI, traumatic brain injury; NTBI, non-traumatic brain injury; 6MWT, six-minute walk test; HRQoL, health-related quality of life; LPA, light physical activity; MVPA, moderate-to-vigorous physical activity.

Data are presented as mean ± SD (range).

**Table 2:** QOLIBRI questionnaire scores by section, total mean, and scaled adjustment.

QOLIBRI section	Mean score
Part A: cognition (7 questions)	3.1 ± 0.9 (1.4–5.0)
Part B: emotions (7 questions)	2.9 ± 0.8 (1.3–4.7)
Part C: daily autonomy (7 questions)	3.1 ± 0.9 (1.6–4.9)
Part D: social relationships (6 questions)	3.3 ± 1.0 (1.5–5.0)
Part E: mental impairments (5 questions)	3.4 ± 0.8 (2.2–5.0)
Part F: physical impairments (5 questions)	3.1 ± 0.8 (1.2–4.2)
Total mean	3.1 ± 0.8 (1.8–4.3)
Adjusted HRQoL (out of 100)	53.4 ± 15.0 (20.9–82.8)

Data are presented as mean ± SD (range). The QOLIBRI questionnaire responses for Parts A–D were scored numerically (e.g., “not at all” = 0, “very” = 5). QOLIBRI responses for Parts E and F were inversely scored (e.g., “not at all” = 5, “very” = 0). The mean score for each section was calculated (e.g., sum of section A values/number of questions in section A). The six-section means (A–F) were then averaged to calculate a total mean score (sum of section mean scores/6). The total mean (e.g., 3.8 out of 5) was converted to a 0–100 scale (0 = no HRQoL, 100 = perfect HRQoL). This conversion was done by subtracting one from the total mean and multiplying by 25 [e.g., (3.8 − 1) × 25 = 70].

**Figure 1:**
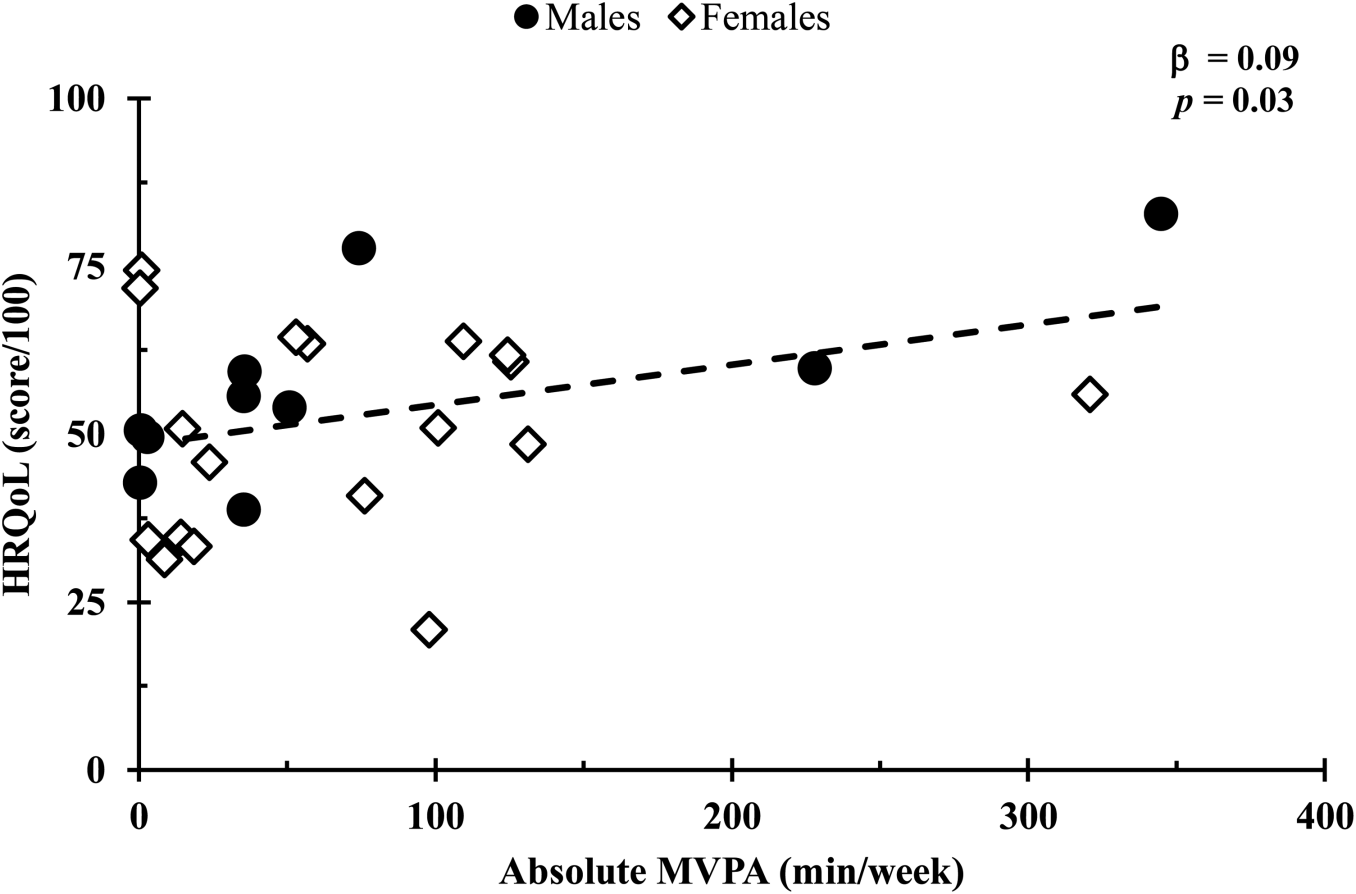
Comparison of the absolute MVPA duration with HRQoL scores. Relationships were determined using multiple regression, with a covariate-adjusted model including age and 6-min walk test distance. Circles represent males, while diamonds represent females. Data are presented for *n* = 26.

**Figure 2:**
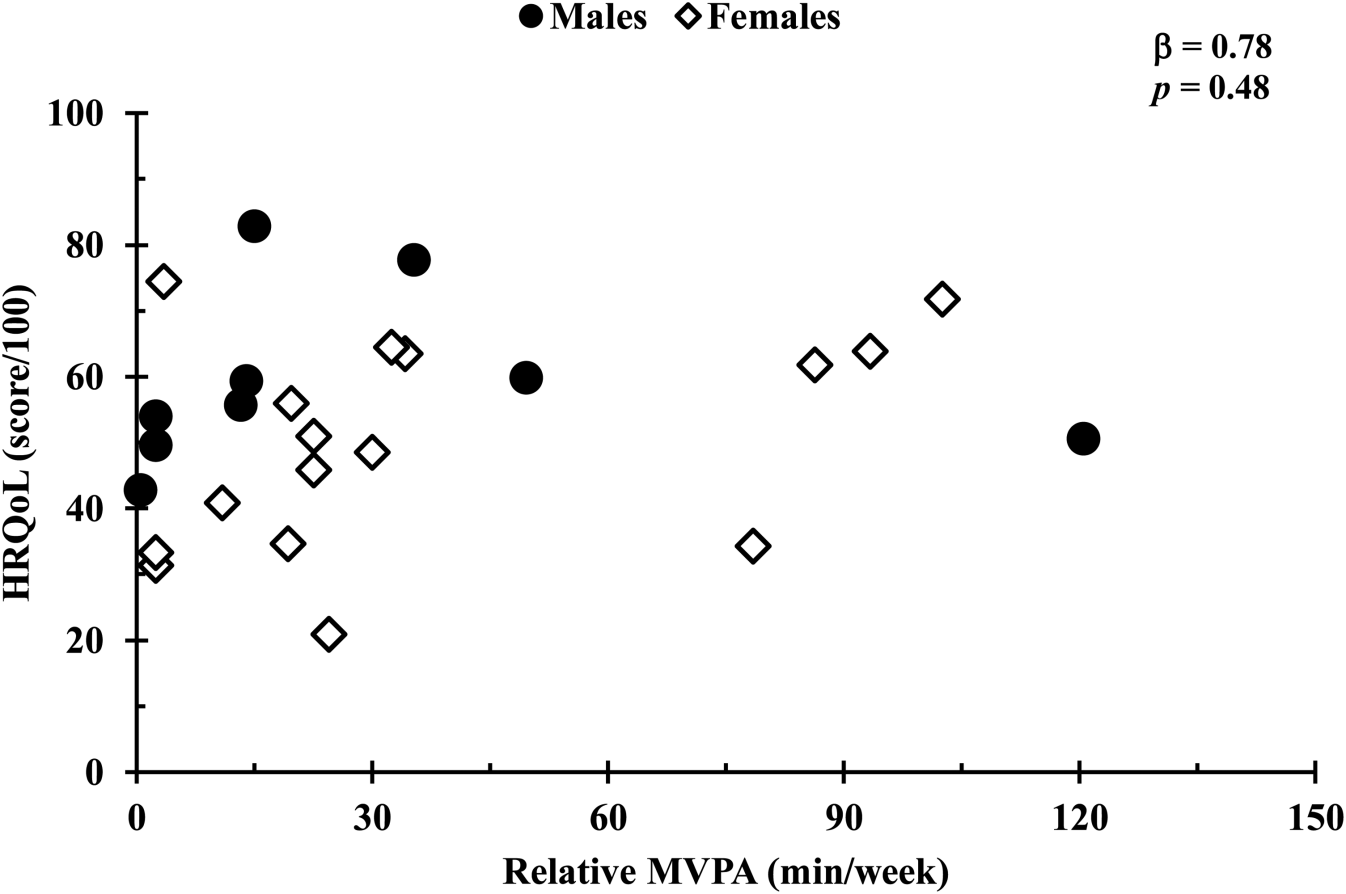
Comparison of relative MVPA duration and HRQoL scores. Relationships were determined using linear regression, with a covariate-adjusted model including age. Circles represent males, while diamonds represent females. Data are presented for *n* = 26.

In a covariate-adjusted model that considered age and 6MWT distance, absolute MVPA was positively associated with HRQoL (*β* = 0.09, *p* *=* 0.03; Figure 1), whereas total, LPA, and step count (all *p*’s *=* 0.24) were not. In addition, HRQoL was not associated with relative physical activity for total, LPA, or MVPA (all *p* values s > 0.14; Figure 2). Also, no associations were observed between standing time or sedentary time and HRQoL (both *p* values > 0.95).

## Discussion

The purpose of this study was to examine the relationship between physical activity and HRQoL in outpatients with ABI. Consistent with our hypothesis, more time spent in absolute MVPA was associated with increased HRQoL independent of age and 6MWT. However, no associations were observed for either relative physical activity or absolute total physical activity. The findings of this study support the beneficial impact of absolute MVPA on HRQoL in individuals with ABI who have habitually low HRQoL (scored <60/100) (25).

Physical activity is defined as any movement produced by skeletal muscles that increases energy expenditure above resting levels (35). It is recommended by both the CSEP and the ACSM that physical activity intensities be individualized based on factors such as aerobic capacity or physical functioning, affecting those with both high and low fitness levels (20, 21). Our study demonstrated that absolute MVPA was associated with better HRQoL, and there was no association for any intensity of relative physical activity. These findings differ from the CSEP and ACSM organizational recommendations, suggesting that a sufficient *absolute* intensity of movement may be needed to improve HRQoL rather than scaling MVPA to individual fitness. Future work is needed to determine the most effective activity interventions for this population. Considering that individuals with ABI typically do not meet guideline recommendations for physical activity duration (13) and have much lower average HRQoL scores than the general population (9), this research indicates that increasing the amount of physical activity to meet absolute thresholds of MVPA (e.g., brisk walking, cycling) may be an effective strategy to improve HRQoL in individuals with ABI.

For individuals with ABI, physical activity is an effective method to improve cognitive function (36), cardiovascular fitness (37), and overall mood (38). The QOLIBRI uses a threshold score of <60/100 to indicate impaired HRQoL (25). The majority of our sample (77%) reported average values of <60, with a total sample average of 53.4/100. Due to the variety of factors that encompass HRQoL (mental, physical, and emotional domains), there may be several mechanisms underpinning the association of MVPA with HRQoL. Increased MVPA has been shown to decrease bodily pain and can help reduce chronic pain (39, 40). Regular physical activity promotes pain relief by reducing N-methyl-D-aspartate receptor phosphorylation, reducing serotonin transporter expression, and increasing serotonin levels (40). In addition, increased MVPA has also been associated with improved physical functioning (41), including increased muscle strength, aerobic capacity, and bone density, which are closely linked with HRQoL (42). Increased physical activity is associated with improved overall mood (38), reductions in anxiety and depression (43), exercise-induced neurogenesis (44), and improved self-efficacy (45). In addition, HRQoL may be impacted by the social aspect of exercise, as increased social activity is associated with higher HRQoL (46, 47). This effect may also work collectively when exercise is conducted outdoors, as spending more time outdoors has been shown to improve self-rated health and reduce negative emotions (48). The factors linking MVPA and HRQoL are likely to be multi-factorial, spanning the emotional, mental, and physical domains. Future research should explore absolute-intensity MVPA intervention models in clinical populations and investigate the mechanistic underpinnings of improved HRQoL.

The primary limitation of the study is its cross-sectional design, which prevents the determination of causality. However, this work is important for directing future intervention studies and applies novel, disease-specific HRQoL outcomes, objective habitual activity monitoring, and consideration of relative activity thresholds. Our results may not be extrapolated to the general population, as this study implemented an ABI-specific scale of HRQoL in outpatients diagnosed with ABI. In addition, our sample consisted of outpatients who could walk without assistance; thus, our results may not apply to individuals with ABI who have acute injuries or mobility challenges. Furthermore, maximal aerobic fitness was estimated based on a published equation for 6MWT (27), rather than directly measured with a VO_2max_ test. However, our observations provide valuable insight into a relatively understudied perspective regarding the most optimal physical activity intensity and threshold for those with ABI.

In outpatients with ABI, who generally report impaired HRQoL, engaging in more absolute-intensity MVPA was associated with better HRQoL. Strategies that investigate and promote the impact of more absolute MVPA may be beneficial lifestyle behaviors that improve HRQoL.

## Data Availability

The raw data supporting the conclusions of this article will be made available by the authors upon reasonable request.

## References

[1] Jones TM Dean CM Hush JM Dear BF Titov N. A systematic review of the efficacy of self-management programs for increasing physical activity in community-dwelling adults with acquired brain injury (ABI). Syst Rev. (2015) 4:51. 10.1186/s13643-015-0039-x 25927591 PMC4422226

[2] Goldman L Siddiqui EM Khan A Jahan S Rehman MU Mehan S Understanding acquired brain injury: a review. Biomedicines. (2022) 10:2167. 10.3390/biomedicines10092167 36140268 PMC9496189

[3] Giustini A Pistarini C Pisoni C. Traumatic and nontraumatic brain injury. Handb Clin Neurol. (2013) 110:401–9. 10.1016/B978-0-444-52901-5.00034-4 23312659

[4] Ponsford JL Downing MG Olver J Ponsford M Acher R Carty M Longitudinal follow-up of patients with traumatic brain injury: outcome at two, five, and ten years post-injury. J Neurotrauma. (2014) 31:64–77. 10.1089/neu.2013.2997 23889321

[5] Revicki DA Kleinman L Cella D. A history of health-related quality of life outcomes in psychiatry. Dialogues Clin Neurosci. (2014) 16:127–35. 10.31887/DCNS.2014.16.2/drevicki 25152652 PMC4140507

[6] Yin S Njai R Barker L Siegel PZ Liao Y. Summarizing health-related quality of life (HRQOL): development and testing of a one-factor model. Popul Health Metr. (2016) 14:22–32. 10.1186/s12963-016-0091-3 27408606 PMC4940947

[7] Gerber GJ Gargaro J McMackin S. Community integration and health-related quality-of-life following acquired brain injury for persons living at home. Brain Inj. (2016) 30:1552–60. 10.1080/02699052.2016.1199896 27564085

[8] von Steinbüchel N Meeuwsen M Zeldovich M Vester JC Maas A Koskinen S Differences in health-related quality of life after traumatic brain injury between varying patient groups: sensitivity of a disease-specific (QOLIBRI) and a generic (SF-36) instrument. J Neurotrauma. (2020) 37:1242–54. 10.1089/neu.2019.6627 31801408

[9] Åkerlund E Sunnerhagen KS Persson HC. Fatigue after acquired brain injury impacts health-related quality of life: an exploratory cohort study. Sci Rep. (2021) 11:221–53. 10.1038/s41598-021-01617-4 34773047 PMC8590006

[10] Ross R Chaput J-P Giangregorio LM Janssen I Saunders TJ Kho ME Canadian 24-hour movement guidelines for adults aged 18–64 years and adults aged 65 years or older: an integration of physical activity, sedentary behaviour, and sleep. Appl Physiol Nutr Metabolism. (2020) 45:S57–102. 10.1139/apnm-2020-0467 33054332

[11] Feng H Yang L Liang YY Ai S Liu Y Liu Y Associations of timing of physical activity with all-cause and cause-specific mortality in a prospective cohort study. Nat Commun. (2023) 14:930. 10.1038/s41467-023-36546-5 36805455 PMC9938683

[12] Fernandes RM Correa MG dos Santos MAR Almeida APCPSC Fagundes NCF Maia LC The effects of moderate physical exercise on adult cognition: a systematic review. Front Physiol. (2018) 9:667. 10.3389/fphys.2018.00667 29937732 PMC6002532

[13] Rand D Eng JJ Tang P-F Jeng J-S Hung C. How active are people with stroke? Use of accelerometers to assess physical activity. Stroke. (2009) 40:163–8. 10.1161/STROKEAHA.108.523621 18948606

[14] English C Manns PJ Tucak C Bernhardt J. Physical activity and sedentary behaviors in people with stroke living in the community: a systematic review. Phys Ther. (2014) 94:185–96. 10.2522/ptj.20130175 24029302

[15] Rand D Eng JJ Tang P-F Hung C Jeng J-S. Daily physical activity and its contribution to the health-related quality of life of ambulatory individuals with chronic stroke. Health Qual Life Outcomes. (2010) 8:80. 10.1186/1477-7525-8-80 20682071 PMC2927504

[16] Pérez-Rodríguez M Gutiérrez-Suárez A Arias JÁR Andreu-Caravaca L Pérez-Tejero J. Effects of exercise programs on functional capacity and quality of life in people with acquired brain injury: a systematic review and meta-analysis. Phys Ther. (2022) 103:pzac153. 10.1093/ptj/pzac153 36336977

[17] Pérez-Rodríguez M García-Gómez S Coterón J García-Hernández JJ Pérez-Tejero J. Physical activity and sport for acquired brain injury (PASABI): a non-randomized controlled trial. Medicina. (2021) 57:122. 10.3390/medicina57020122 33572946 PMC7911011

[18] Martin S Mooruth D Guerdoux-Ninot E Mazzocco C Brouillet D Taconnat L Demographic characteristics, motivation and perception of change as determinants of memory compensation self-reports after acquired brain injury. Front Psychol. (2021) 12:607035. 10.3389/fpsyg.2021.607035 34335350 PMC8318033

[19] Jetté M Sidney K Blümchen G. Metabolic equivalents (METS) in exercise testing, exercise prescription, and evaluation of functional capacity. Clin Cardiol. (1990) 13:555–65. 10.1002/clc.4960130809 2204507

[20] American College of Sports Medicine. ACSM'S Guidelines for Exercise Testing and Prescription. Philadelphia, PA: Wolters Kluwer Health (2013). 10.1249/JSR.0b013e31829a68cf 23851406

[21] Canadian Society of Exercise Physiology. CSEP—Physical Activity Training for Health (PATH), 3rd ed. Ottawa, ON: CSEP (2021).

[22] Kujala UM Pietila J Myllymaki T Mutikainen S Fohr T Korhonen I Physical activity: intensity versus relative-to-fitness-level volumes. Med Sci Sports Exerc. (2017) 49:474–81. 10.1249/MSS.0000000000001134 27875497

[23] Mossberg KA Ayala D Baker T Heard J Masel B. Aerobic capacity after traumatic brain injury: comparison with a nondisabled cohort. Arch Phys Med Rehabil. (2007) 88:315–20. 10.1016/j.apmr.2006.12.006 17321823

[24] Faul F Erdfelder E Buchner A Lang A-G. Statistical power analyses using G*Power 3.1: tests for correlation and regression analyses. Behav Res Methods. (2009) 41:1149–60. 10.3758/BRM.41.4.1149 19897823

[25] Wilson L Marsden-Loftus I Koskinen S Bakx W Bullinger M Formisano R Interpreting quality of life after brain injury scores: cross-walk with the short form-36. J Neurotrauma. (2017) 34:59–65. 10.1089/neu.2015.4287 27297289

[26] Mossberg KA Fortini E. Responsiveness and validity of the six-minute walk test in individuals with traumatic brain injury. Phys Ther. (2012) 92:726–33. 10.2522/ptj.20110157 22282772 PMC3345338

[27] Šagát P Kalčik Z Bartik P Šiška Ľ Štefan L. A simple equation to estimate maximal oxygen uptake in older adults using the 6 min walk test, sex, age and body mass index. J Clin Med. (2023) 12:4476. 10.3390/jcm12134476 37445511 PMC10342654

[28] O’Brien MW Wu Y Petterson JL Bray NW Kimmerly DS. Validity of the activPAL monitor to distinguish postures: a systematic review. Gait Posture. (2022) 94:107–13. 10.1016/j.gaitpost.2022.03.002 35276456

[29] Wu Y Petterson JL Bray NW Kimmerly DS O’Brien MW. Validity of the activPAL monitor to measure stepping activity and activity intensity: a systematic review. Gait Posture. (2022) 97:165–73. 10.1016/j.gaitpost.2022.08.002 35964334

[30] Hart TL Swartz AM Cashin SE Strath SJ. How many days of monitoring predict physical activity and sedentary behaviour in older adults? Int J Behav Nutr Phys Act. (2011) 8:62. 10.1186/1479-5868-8-62 21679426 PMC3130631

[31] Edwardson CL Winkler EAH Bodicoat DH Yates T Davies MJ Dunstan DW Considerations when using the activPAL monitor in field-based research with adult populations. J Sport Health Sci. (2017) 6:162–78. 10.1016/j.jshs.2016.02.002 30356601 PMC6188993

[32] Johns JA Frayne RJ Goreham JA Kimmerly DS O’Brien MW. The bout cadence method improves the quantification of stepping cadence in free-living conditions. Gait Posture. (2020) 79:96–101. 10.1016/j.gaitpost.2020.04.014 32387810

[33] O’Brien MW Kivell MJ Wojcik WR D’Entremont GR Kimmerly DS Fowles JR. Influence of anthropometrics on step-rate thresholds for moderate and vigorous physical activity in older adults: scientific modeling study. JMIR Aging. (2018) 1:e12363. 10.2196/12363 31518246 PMC6715008

[34] Seefeldt V Malina RM Clark MA. Factors affecting levels of physical activity in adults. Sports Med. (2002) 32:143–68. 10.2165/00007256-200232030-00001 11839079

[35] Caspersen CJ Powell KE Christenson GM. Physical activity, exercise, and physical fitness: definitions and distinctions for health-related research. Public Health Rep. (1985) 100:126–31. 3920711 PMC1424733

[36] Grealy MA Johnson DA Rushton SK. Improving cognitive function after brain injury: the use of exercise and virtual reality. Arch Phys Med Rehabil. (1999) 80:661–7. 10.1016/S0003-9993(99)90169-7 10378492

[37] Mossberg KA Amonette WE Masel BE. Endurance training and cardiorespiratory conditioning after traumatic brain injury. J Head Trauma Rehab. (2010) 25:173–83. 10.1097/HTR.0b013e3181dc98ff PMC2885899 20473091

[38] Driver S Ede A. Impact of physical activity on mood after TBI. Brain Inj. (2009) 23:203–12. 10.1080/02699050802695574 19205956

[39] Kaleth AS Saha CK Jensen MP Slaven JE Ang DC. Effect of moderate to vigorous physical activity on long-term clinical outcomes and pain severity in fibromyalgia. Arthritis Care Res (Hoboken). (2013) 65:1211–8. 10.1002/acr.21980 23401486 PMC3672379

[40] Lima LV Abner TSS Sluka KA. Does exercise increase or decrease pain? Central mechanisms underlying these two phenomena. J Physiol. (2017) 595:4141–50. 10.1113/JP273355 28369946 PMC5491894

[41] Yatsugi H Chen T Chen S Liu X Kishimoto H. The associations between objectively measured physical activity and physical function in community-dwelling older Japanese men and women. Int J Environ Res Public Health. (2022) 19:369. 10.3390/ijerph19010369 PMC8744806 35010628

[42] Manini TM Pahor M. Physical activity and maintaining physical function in older adults. Br J Sports Med. (2008) 43:28–31. 10.1136/bjsm.2008.053736 18927164 PMC3104323

[43] Paluska SA Schwenk TL. Physical activity and mental health. Sports Med. (2000) 29:167–80. 10.2165/00007256-200029030-00003 10739267

[44] Ernst C Olson AK Pinel JPJ Lam RW Christie BR. Antidepressant effects of exercise: evidence for an adult-neurogenesis hypothesis? J Psychiatry Neurosci. (2006) 31:84–92. 16575423 PMC1413959

[45] Miller KJ Mesagno C McLaren S Grace F Yates M Gomez R. Exercise, mood, self-efficacy, and social support as predictors of depressive symptoms in older adults: direct and interaction effects. Front Psychol. (2019) 10:2145. 10.3389/fpsyg.2019.02145 31632315 PMC6761306

[46] Busse AL Gil G Santarém JM Filho WJ. Physical activity and cognition in the elderly: a review. Dement Neuropsychol. (2009) 3:204–8. 10.1590/S1980-57642009DN30300005 29213629 PMC5618974

[47] Park HK Chun SY Choi Y Lee SY Kim SJ Park E-C. Effects of social activity on health-related quality of life according to age and gender: an observational study. Health Qual Life Outcomes. (2015) 13:140. 10.1186/s12955-015-0331-4 26361977 PMC4566195

[48] Twohig-Bennett C Jones A. The health benefits of the great outdoors: a systematic review and meta-analysis of greenspace exposure and health outcomes. Environ Res. (2018) 166:628–37. 10.1016/j.envres.2018.06.030 29982151 PMC6562165

